# Clinicopathological difference between gastric cancer in the lesser curvature and gastric cancer in the greater curvature

**DOI:** 10.1097/MD.0000000000029984

**Published:** 2022-08-19

**Authors:** Guo-Cai Li, Hong-Wei Zhang, Hong-Gang Tian, Yun Zhao, Qin-Xian Huang, Ze-Yu Xu, Xiao-Hui Xi, Kai Zhang

**Affiliations:** a Division of Digestive Surgery, Hospital of Digestive Diseases, Xi’an International Medical Centre, Xi’an, Shaanxi Province, China.; b Division of Digestive Surgery, Xijing Hospital of Digestive Diseases, Fourth Military Medical University, Xi’an, Shaanxi Province, China.

**Keywords:** clinicopathological characteristic, gastric cancer, larger curvature, lesser curvature

## Abstract

Gastric cancer (GC) is a heterogeneous disease; the tumor distribution and molecular subtype could affect the prognosis of patients with GC. However, the clinicopathological difference between GC in the lesser and that in the greater curvature remains unknown. In this study, we aimed to investigate the difference and provide new clues for the treatment of GC. Between January 2010 and August 2014, 1249 consecutive patients with GC in the lesser or greater curvature were treated in our surgery department; data related to the demographic characteristics, pathological type, tumor grade, tumor size, TNM stage, tumor markers, operative methods, complications, and follow-up were retrospectively analyzed using a univariate analysis and the Kaplan–Meier method. The tumor size in lesser curvature was larger than that in the greater curvature (4.95 ± 2.57 vs 4.43 ± 2.62 cm, *P* = .034); patients with GC in the lesser curvature had a higher incidence of total gastrectomy and a lower incidence of distal gastrectomy than those with GC in the greater curvature (60.2% vs 43.2%, and 34.8% vs 49.2%, *P* = .002). No significant differences were found in the 5-year survival rate between patients with GC in the greater curvature and those with GC in the lesser curvature (62.6% vs 66.1%, *P* = .496). The epidermal growth factor receptor (EGFR) expression rate of tumors in the lesser curvature was 40.55%, which was significantly higher than that of tumors in the greater curvature (25.92%, *P* = .024), while the 5-year survival rate of patients with EGFR-positive expression was 50.8%, which was significantly lower than that of patients with EGFR-negative expression (64.8%, *P* = .021). Significant differences were observed in the clinicopathological features between GC in the lesser curvature and that in the greater curvature. These differences contribute to the improvement in the treatment outcome.

## 1. Introduction

Gastric cancer (GC) is the third most common type of cancer and second leading cause of mortality worldwide. Although the number of deaths has decreased, 1.3 million cases of GC and 819,000 deaths have been reported in 2015.^[1]^ The 5-year survival of GC is 25% to 39%,^[2]^ while the median survival times are 50, 14, and 3 months for patients who received chemotherapy plus surgery, patients who received chemotherapy alone, and patients who received the best supportive care, respectively.^[3]^

GC is a heterogeneous disease. The Cancer Genome Atlas project has proposed a molecular classification for gastric adenocarcinomas.^[4]^ A tissue subtype of gastric adenocarcinomas was also established by Birkman et al.^[5]^ Through these molecular and tissue classification, the distribution of molecular subtypes in GC was determined. Epstein–Barr virus–positive intestinal-type tumors are more frequently found in the gastric corpus. Intestinal-type tumors with TP53 aberrations are commonly detected in the proximal gastric area. These findings made it feasible to integrate genome-based diagnostic and therapeutic methods.^[6–8]^

The different distributions of molecular subtypes further suggest that there are clinicopathological differences in the tumor distribution, which could affect the prognosis of GC. The incidence of cardia and fundus cancer remained the same in recent years, but that of corpus cancer increased. In addition, the proportion of localized tumors in GC increased, but that of regional tumor decreased.^[9]^ Moreover, microsatellite-unstable tumors occurring in the antrum have the best prognosis and the lowest frequency of recurrence.^[10]^

The lesser curvature near the angular incisure of the stomach is the most common site of GC, while the incidence of tumor development in the greater curvature is <3%.^[11]^ To date, the clinicopathological difference between GC in the lesser curvature and GC in the greater curvature remains unclear. Hence, this study aimed to investigate this difference and provide useful clues for the proper treatment of GC.

## 2. Methods

### 2.1. Patient enrollment

Between January 2010 and August 2014, 4421 consecutive patients with GC underwent gastrectomy in the First Department of Digestive Surgery of XiJing Hospital, Fourth Military Medical University, Xi’an, China. In this retrospective cohort study, all patient data were evaluated by 2 researchers. Patients who were diagnosed with gastric adenocarcinoma according to their pathologic characteristics; who underwent gastrectomy or explorative surgery and whose tumors were located at the lesser or greater curvatures and were clearly recorded by the surgeons; and with no severe primary disease and with an American Society Anesthesiology Physical Status Classification of I or II were included in the study.

This study was approved by the Ethics Committee of the Fourth Military Medical University. All patients received verbal and written information regarding the study and provided informed consent prior to surgery.

### 2.2. Demographic and preoperative data

The demographic data, including sex, age, history of past illness, and preoperative data, were collected to analyze the comparability of the groups.

### 2.3. Expression level of the serum markers for GC

The levels of tumor markers, carcinoembryonic antigen (CEA), carbohydrate antigen (CA)19-9, alpha fetoprotein, and CA125 were measured in our hospital using the radioimmune method, and 5 ng/mL, 7 ng/mL, 27 U/mL, and 35, U/mL were assigned as cutoff values, respectively; furthermore, the expression level of tumor markers was classified as either positive or negative.

### 2.4. Immunohistochemistry

Several tumor markers have been confirmed to be closely related to the clinicopathologic characteristics and prognosis of GC and are commonly used in the current clinic, including epidermal growth factor receptor (EGFR) and human epidermal growth factor receptor-2 (HER-2).^[12–14]^ These markers were evaluated postoperatively through immunohistochemical staining. Two pathologists independently observed and interpreted the results of the immunohistochemical staining.

### 2.5. Perioperative observations

The patients underwent radical gastrectomy with D2 lymphadenectomy using either the laparotomy or laparoscopic method. The extent of resection of the stomach (total, proximal, or distal gastrectomy) was determined based on the tumor size, location, infiltration of organ, and pathological type. When tumor infiltration in the surrounding organ was noted, an enlarged gastrectomy combined with organ resection was performed; when profound tumor metastasis was noted, an explosive or palliative operation was performed. Anastomoses, including esophagogastrostomy, gastroduodenostomy, and esophagojejunostomy, were performed using a 28-mm diameter circular stapler. The volume of intraoperative blood loss and operation time were determined by the anesthesiologist.

The postoperative data included pathological type, Borrmann type, grade of differentiation, tumor size, number of intensive care unit stay, and 90-day mortality. The histological subtype and pathological stage were determined using the Union for International Cancer Control and TNM classification for GC. Resection status (R-status) was divided into 3 classifications: R0, a complete resection with negative margins; R1, microscopic residual disease (positive margins); and R2, gross (macroscopic) residual disease.

Postoperative complications, including anastomotic complication, wound infection, wound rupture, lung infection, bleeding, reoperation, duodenal leak, and intestinal obstruction, were evaluated. A water-soluble radiological contrast enema was performed at 6 to 8 days postoperatively to assess for anastomotic leaks. A clinical leak was defined by extravasation of the contrast medium detected on radiography. The complications were evaluated using the Clavien–Dindo classification.^[15]^

### 2.6. Follow-up data

All patients were followed for 5 years after the operation. At the end of follow-up, the status of patients was recorded, which included the number of patients who survived, died, and were lost to follow-up.

### 2.7. Statistical analysis

Statistical analysis was performed using the SPSS 17 software (SPSS Inc., Chicago, IL). To determine the differences among the study groups, the measurement data were analyzed using Student *t* test. The differences in expression rate among the study groups were analyzed using the Pearson chi-square (χ^2^) test. The Fisher exact test was used to assess the differences in the positive rates when the number of total cases was <40. A *P* value of <.05 was considered significant. Survival analysis was performed using the Kaplan–Meier method.

## 3. Results

### 3.1. Baseline characteristics

**Figure 1.**
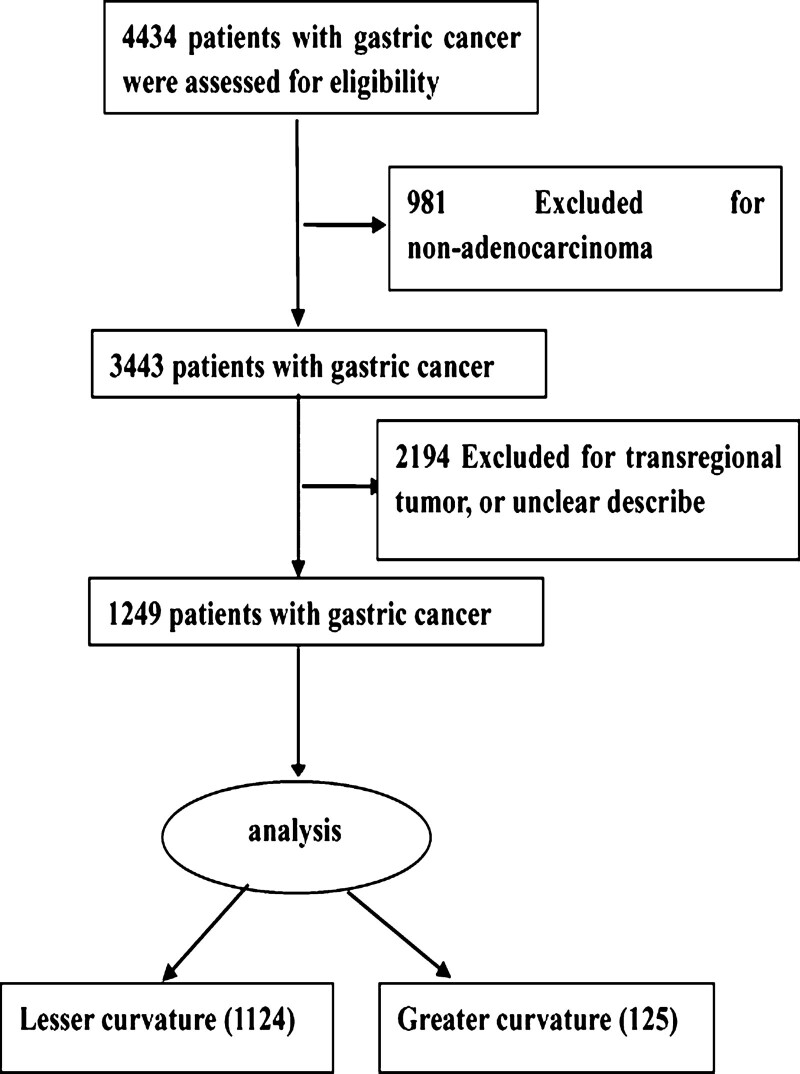
Flow diagram of the data enrollment process.

A total of 1249 patients met the inclusion criteria and were analyzed in this study (Fig. 1). Among them, 1124 patients had GC in the lesser curvature, while 125 patients had GC in the greater curvature. The comparison of baseline data between the group with GC in the lesser curvature and the group with GC in the greater curvature is presented in Table 1. No significant differences were observed between the lesser and greater curvature groups in terms of preoperative variables, such as age and sex. The patients with GC in the lesser curvature had a higher incidence of family history of neoplasm than those with GC in the greater curvature (9.4% vs 4%, *P* = .041; Table 1).

**Table 1:** Pathological characteristics of patients with gastric cancer.

Feature		Gastric cancer		*P* value
		Lesser curvature	Greater curvature	
No. of cases		1124	125	
Male/female		872/252	90/35	.178
Age (yr, mean ± SD)		56.97 ± 10.98	55.92 ± 12.04	.312
Family history of tumor (%)		106 (9.4)	5 (4)	.041
Heart disease (%)		21 (1.9)	6 (4.8)	.046
Hypertension (%)		133 (11.8)	18 (14.4)	.389
Others (%)		130 (11.6)	14 (11.2)	.887
Tumor size (cm)		4.95 ± 2.57	4.43 ± 2.62	.034
Borrman type	I	37 (5.6)	4 (5.6)	.817
	II	211 (31.9)	26 (36.6)	
	III	307 (46.5)	27 (38.1)	
	IV	105 (15.9)	14 (19.7)	
Grade of differentiation	Well	119 (17.3)	19 (23.8)	.350
	Moderate	300 (43.6)	31 (38.7)	
	Poor	269 (39.1)	30 (37.5)	
TNM stages (UICC)	I	272 (23.47)	29 (26.61)	.313
	II	139 (11.99)	18 (16.51)	
	III	508 (43.83)	39 (35.78)	
	IV	240 (20.71)	23 (21.1)	
LVI	No	772 (68.7)	92 (73.6)	.259
	Yes	352 (31.3)	33 (27.4)	
Lymph node harvest (Mean ± SD)		16.86 ± 9.94	17.77 ± 12.02	.642
Lauren histologic type	Intestinal	714 (63.5)	76 (60.8)	.549
	Diffuse	410 (36.5)	49 (39.2)	
R-status	R0	1063 (96.1)	111 (91.7)	.025
	R1	43 (3.9)	10 (8.3)	
	R2	0 (0)	0 (0)	
Adjuvant/neoadjuvant chemotherapy	No	733 (59.9)	84 (67.2)	.693
	Yes	390 (40.1)	41 (32.8)	

Student *t* test was used to analyze measurement data. Chi-square test and the Fisher exact test were used to analyze categorical variables.

LVI = lymphovascular invasion, R-status = a complete resection with negative margins (R0 resection), R1 = microscopic residual disease (positive margins), R2 = gross (macroscopic) residual disease, SD = standard deviation, UICC = union for international cancer control.

No significant difference was observed in the pathological type, histological subtype, Borrmann type, tumor differentiation, and TNM stage between patients with GC in the greater curvature and those with GC in the lesser curvature. Meanwhile, the tumor found in the lesser curvature was larger than that in the greater curvature (4.95 ± 2.57 vs 4.43 ± 2.62 cm, *P* = .034). In addition, the incidence of R0 resection in patients with GC in the lesser curvature was higher than in those with GC in the greater curvature (96.1% vs 91.7%, *P* = .025; Table 1).

### 3.2. Comparison of tumor markers of GC

The levels of tumor markers, such as CEA, CA19-9, CA125, and alpha fetoprotein, were not significantly different between patients with GC in the lesser curvature and those with GC in the greater curvature. According to cutoff values, no significant difference was observed in the positive rate of these markers (Table 2).

**Table 2:** The expression level of serum markers in patients with gastric cancer.

		Gastric cancer		*P* value
		Lesser curvature	Great curvature	
CEA	(ng/mL)	14.70 ± 118.48	11.47 ± 79.69	.780
	Total no.	974	110	
	n (%)	165 (16.9%)	13 (11.8%)	.370
CA19-9	(U/L)	77.72 ± 473.35	141.89 ± 609.27	.213
	Total no.	908	100	
	n (%)	165 (18.2%)	22 (22.0%)	.438
AFP	(ng/mL)	17.67 ± 167.89	18.51 ± 117.03	.961
	Total no.	911	99	
	n (%)	66 (7.2%)	12 (12.1%)	.225
CA125	(U/L)	15.69 ± 34.40	16.45 ± 36.79	.832
	Total no.	922	103	
	n (%)	42 (4.6%)	4 (3.9%)	.639

The measurement data were described as mean ± standard deviation and were analyzed by Student *t* test. Total no. mean the total specimen detected; n (%) denoted positive specimen and rate. Chi-square test and the Fisher exact test were used to analyze categorical variables.

AFP = alpha fetoprotein, CA = carbohydrate antigen, CEA = carcinoembryonic antigen.

### 3.3. Comparison of operative data

The patients with GC in the lesser curvature had a higher incidence of total gastrectomy, but a lower incidence of distal gastrectomy than those with GC in the greater curvature (60.2% vs 43.2%, and 34.8% vs 49.2%, *P* = .002). In addition, the incidence of gastrectomy with combined resection of other organs was lower in patients with GC in the lesser curvature than in those with GC in the greater curvature (10.6% vs 20.8%, *P* = .001). The main organs resected along with the stomach in patients with GC in the lesser curvature were the gallbladder, left liver lobe, and spleen, while those with GC in the larger curvature were the spleen and pancreas (*P* = .005). Moreover, the amount of intraoperative blood loss in patients with GC in the lesser curvature was lesser than that in patients with GC in the greater curvature (218.23 ± 196.37 vs 272.88 ± 262.27 mL, *P* = .041). However, the operative time was similar between the 2 curvatures. No statistical difference was observed in the number of patients who were admitted in the intensive care unit (1.7% vs 1.6%, *P* = .941); no statistical difference was also observed in the 90-day mortality between the 2 curvatures (2.6% vs 2.4%, *P* = .904; Table 3).

**Table 3:** Comparison of operative data in patients with gastric cancer.

Variables	Lesser curvature	Greater curvature	*P* value
	No.	No.	
Operative methods			
Laparotomy	723 (90.1%)	79 (9.1%)	.892
Laparoscopic	374 (89.9%)	42 (10.1%)	
Gastrectomy methods			
Total	662 (60.2%)	51 (43.2%)	.002
Distal	383 (34.8%)	58 (49.2%)	
Proximal	54 (4.9%)	9 (7.6%)	
Combined resection			
No	1005 (89.4%)	99 (79.2%)	.001
Yes	119 (10.6%)	26 (20.8%)	
Organ combined resection			
Spleen	24 (20.1%)	6 (23.0%)	.005
Pancreas	8 (6.7%)	5 (19.2%)	
Colon	5 (4.2%)	6 (23.1%)	
Liver	8 (6.7%)	0 (0%)	
Gallbladder	66 (55.4%)	8 (30.8%)	
Ovary	2 (1.6%)	0 (0%)	
Others	6 (5.0%)	1 (3.8%)	
Operation time (min)	205.62 ± 87.22	211.84 ± 70.63	.485
Bleeding (mL)	218.23 ± 196.37	272.88 ± 262.27	.041
No. of ICU stay	19 (1.7%)	2 (1.6%)	.941
90-d mortality	29 (2.6%)	3 (2.4%)	.904

Student *t* test was used to analyze age, and chi-square test were used to analyze categorical variables, respectively.

ICU = intensive care unit, SD = standard deviation.

### 3.4. Postoperative complications

None of the patients died during their hospital stay. The incidence of total complications in patients with GC in the lesser curvature was not statistically different from that in patients with GC in the larger curvature (29.98% vs 40%, *P* = .097), and the anastomotic complications were similar between the 2 curvatures (0.71% vs 0%, *P* = .344). In addition, no differences were found in the pulmonary complications, wound rupture, duodenum leak, anastomotic leakage and stricture, Clavien–Dindo classification, and severe bleeding according to results of the univariate analysis (Table 4).

**Table 4:** Postoperative complications in patients with gastric cancer.

Postoperative complication	Lesser curvature	Greater curvature	*P* value
Pulmonary complications	25 (2.22%)	3 (2.4%)	.755
Fever	295 (26.25%)	43 (34.4%)	.052
Wound rupture	9 (0.8%)	3 (2.4%)	.110
Severe bleeding	10 (0.89%)	1 (0.8%)	.919
Intestinal obstruction	19 (1.69%)	4 (3.2%)	.278
Duodenum leakage	2 (0.18%)	0 (0%)	.637
Anastomosis stricture	5 (0.44%)	0 (0%)	.455
Anastomosis leakage	3 (0.27%)	0 (0%)	.563
Anastomotic complication (%)	8 (0.71%)	0 (0%)	.344
Clavien–Dindo classification			
Grade I	262 (23.31%)	38 (30.4%)	.944
Grade II	29 (2.58%)	5 (4%)	
Grade III	27 (2.4%)	3 (2.4%)	
Grade IV	10 (0.89%)	2 (1.6%)	
Grade V	9 (0.8%)	2 (1.6%)	
Any complication (%)	337 (29.98%)	50 (40%)	.097

The data were showed by no. Chi-square test was used to analyze complication incidence.

### 3.5. Five-year survival rate between GC in the greater curvature and GC in the lesser curvature

**Figure 2.**
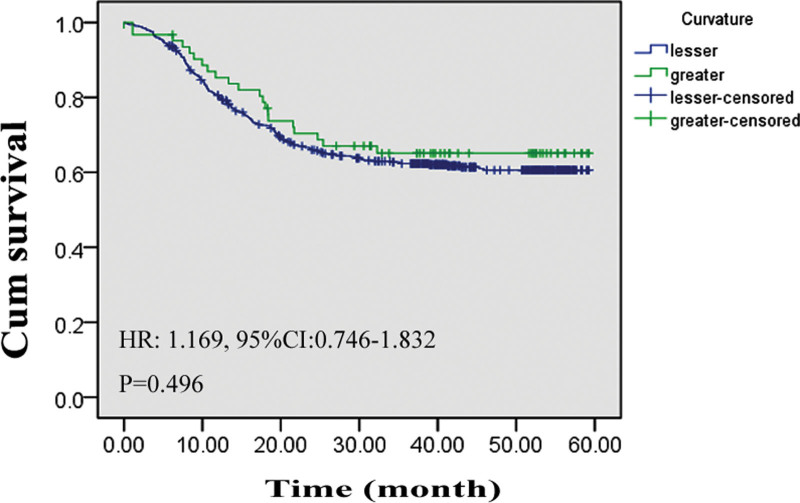
Survival difference between gastric cancer in the lesser curvature and gastric cancer in the larger curvature. Survival curve of patients with gastric cancer located in the lesser curvature and larger curvature. 95% CI = 95% confidence interval, HR = hazard ratio.

A total of 1108 patients had complete follow-up data, and the average flow-up time was 29.14 ± 17.09 months (range: 0.17–66.73 months). No significant difference was observed in the 5-year survival rate between patients with GC in the greater curvature and those with GC in the lesser curvature (62.6% vs 66.1%, 0.496), and the average survival time was not significantly different (43.78 ± 2.78 vs 41.26 ± 0.99 months, *P* = .496). Based on the results of the Kaplan–Meier analysis, the hazard ratio (HR) for death between the 2 curvatures was 1.169 (95% confidence interval [CI]: 0.746–1.832, *P* = .496; Fig. 2). Using Cox regression analysis, we investigated the factors that may affect the survival rate. Age, radical resection effect, TNM stage, and tumor grade of differentiation were closely related to the 5-year survival rate. Meanwhile, the difference in survival rate between patients with GC in the greater curvature and those with GC in the lesser curvature remained unknown (Table 5).

**Table 5:** Factors influencing survival in gastric cancer by Cox regression analysis.

Variable	Regression coefficient	Standard error	OR	*P* value
Sex	−0.374	0.235	0.688	.112
Age	0.022	0.009	1.022	.021
Small/large curvature	0.179	0.333	1.196	.590
R-status	−0.718	0.348	0.488	.039
Organ combined resection	−0.113	0.313	0.893	.717
TNM stage	0.868	0.131	2.381	.000
Adjuvant/neoadjuvant chemotherapy	−0.288	0.204	0.750	.158
Postoperative complication	0.096	0.339	1.100	.778
Grade of differentiation	0.367	0.153	1.443	.016

Sex was coded as 1 = male and 2 = female. Postcomplication was coded as 1 = with complication and 2 = without complication; grade of tumor differentiation was coded as 1 = well, 2 = moderate, 3 = poor, and 4 = no differentiation; TNM stage was coded as 1 = I, 2 = II, 3 = III, and 4 = IV.

OR = odds ratio, R-status = R0 resection indicating complete resection with negative margins, R1 = microscopic residual disease (positive margins), R2 = gross (macroscopic) residual disease.

### 3.6. EGFR expression between GC in the greater curvature and GC in the lesser curvature

**Figure 3.**
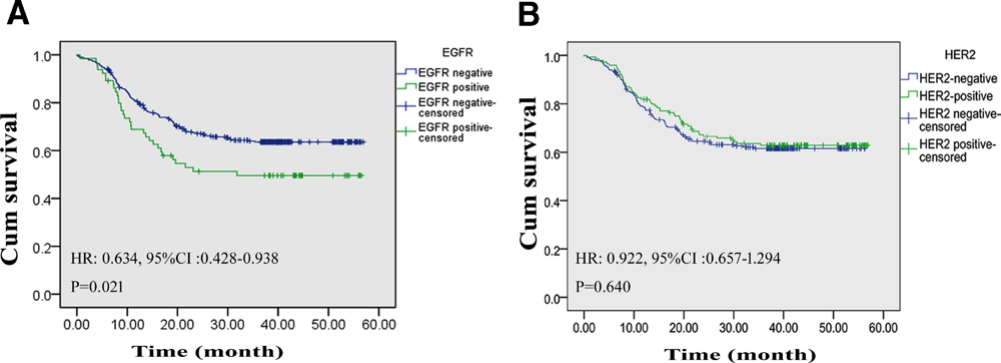
Survival difference according to the expression levels of EGFR. (A) Survival curve of patients with EGFR-positive GC or with EGFR-negative GC. (B) Survival curve of patients with HER-2-positive or with HER2-negative GC. 95% CI = 95% confidence interval, EGFR = epidermal growth factor receptor, GC = gastric cancer, HER-2 = human epidermal growth factor receptor-2, HR = hazard ratio.

The EGFR expression was negatively correlated with the 5-year survival; the survival rate in patients with EGFR-positive expression was 50.8%, which was significantly lower than that in those with EGFR-negative expression (64.8%, *P* = .021); based on the Kaplan–Meier analysis, the HR for death between the 2 groups was 0.634 (95% CI: 0.428–0.938, *P* = .021). The average survival time was also significantly decreased (33.89 ± 2.89 vs 40.90 ± 1.22 months, *P* = .021). However, the HER2 expression rates were not evaluated between the curvatures (36.69% vs 29.25%, *P* = .446; Table 6); the 5-year survival rate was not statistically significant between the HER2-positive group and HER2-negative group (63.8% vs 63.0%, *P* = .640), and the average survival time was not significantly different (41.05 ± 1.76 vs 39.37 ± 1.44 months, *P* = .640); in the Kaplan–Meier analysis, the HR for death between the 2 groups was 0.922 (95% CI: 0.657–01.294, *P* = .640; Fig. 3A, B).

**Table 6:** The expression of EGFR and HER-2 in patients with gastric cancer.

			Gastric cancer		*P* value
		Total	Lesser curvature	Greater curvature	
HER-2	No.	621	556	65	.446
	Positive no. (%)		204 (36.69%)	19 (29.23%)	
EGFR	No.	870	789	81	.024
	Positive no. (%)		320 (40.55%)	21 (25.92%)	

Chi-square test and the Fisher exact test were used to analyze categorical variables.

EGFR = epidermal growth factor receptor, HER-2 = human epidermal growth factor receptor-2.

## 4. Discussion

The present study aimed to investigate clinicopathological difference between GC in the lesser curvature and GC in the greater curvature. Tumor size, the extent of gastrectomy, postoperative complication, and EGFR expression level were significantly different between GC in the lesser curvature and GC in the greater curvature.

In this study, GC more commonly developed in the lesser curvature than in the larger curvature. Our results agreed with those of a previous study, which used a proteomics-based approach.^[11]^ It reported that *Helicobacter pylori* infection is a definite environmental risk factor for the development of GC^[16]^; in addition, the scores of mean atrophy and intestinal metaplasia, which are caused by *H pylori* infection, were higher in patients with GC in the lesser curvature than in those with GC in the greater curvature.^[17]^ Moreover, yellowish-white nodules are commonly observed in patients with *H pylori*-associated gastritis; about 20% of nodules are detected in the corpus mucosa of the lesser curvature, while only 0.9% are detected in the greater curvature.^[18]^ Gastric “‘crawling-type’” adenocarcinoma, a neoplasm histologically comprising irregularly fused glands with low-grade cellular atypia, frequently developed in the lesser curvature of the middle-third of the stomach.^[19]^ Combined with the above studies, our results emphasized the increased cancer susceptibility of the lesser curvature of the stomach. We found a higher ratio of family history in patients with lesser curvature tumor; this finding suggests that GC in the lesser curvature was more frequently correlated with heredity compared with GC in the larger curvature.

The anatomical location-based classification of lymph node metastasis is an important tool for GC prognosis,^[20]^ and the incidence of lymph nodular metastasis tended to be higher in patients with GC in the lower location than in those with GC in the middle/upper location.^[21]^ Therefore, some authors hypothesize that GC in the lesser curvature can be treated using a modified D2 lymphadenectomy; for GC in the greater curvature, a D1(+) lymphadenectomy including lymph nodes nos. 7 and 9 is preferred.^[22]^ In this study, no difference was observed in the lymph node metastasis status between patients with GC in the greater curvature and those with GC in the lesser curvature. Our result was consistent with that of a previous study, which demonstrated that tumor metastasis was similar between the 2 curvatures^[23]^; however, this finding was not in agreement with that of another study, which showed that tumor metastasis frequently occurred in the lower third of the lesser curvature.^[24]^

We investigated the operative mode of gastrectomy in all patients and found that patients with GC in the lesser curvature had a higher incidence of total gastrectomy, but a lower incidence of distal gastrectomy than those with GC in the greater curvature. In addition, the incidence of radical resection was higher in patients with GC in the lesser curvature than in those with GC in the greater curvature. Compared with those with GC in the lesser curvature, patients with GC in the greater curvature had a higher incidence of organ combined resection and a larger volume of intraoperative blood loss. These results suggest that tumor in the greater curvature more commonly infiltrate the adjacent organs such as pancreas and spleen.

In this study, the incidence of total postoperative complications and anastomotic complications was not different between the 2 curvature tumors. Our results were not consistent with those of Hirota and Kim, who reported that operation for lesser curvature tumor has significantly higher risk of postoperative complications than that for greater curvature,^[25]^ such as prolonged abdominal symptoms, food residue, and perforation.^[26]^

Several studies have reported that the 5-year overall survival rate of GC is influenced by tumor size, depth of invasion, lymph node metastasis, chemotherapy, early detection, and radical resection.^[27,28]^ In this study, we found that age, radical resection effect, TNM stage, and tumor grade of differentiation were closely related to the 5-year survival rate; in addition, the incidence of R0 and R1 resection in patients with GC in the lesser curvature was higher than that in patients with GC in the greater curvature. Combined with other studies, our results also emphasized that R0 and R1 have important prognostic information and was consistent with those of another study.^[29]^ However, no significant difference was observed between the GCs in the 2 curvatures based on the Cox regression analysis, and our results were not consistent with the reports of a previous study, which showed worse survival in patients with GC in the greater curvature compared with patients with GC in the lesser curvature.^[30]^

In our study, we investigated the expression status of several tumor markers, which are commonly used in the current clinic.^[31]^ Patients with GC in the lesser curvature had higher levels of EGFR expression compared with those with GC in the larger curvature; in addition, the 5-year survival in EGFR-negative group was significantly higher than that in the EGFR-positive group. Except for EGFR expression, other tumor markers were not statistically different between the 2 curvatures. The enhancement of EGFR in the lesser curvature tumor may indicate its new prognostic value and provide new clues for GC treatment.

Our study has some limitations. All patients included in this study underwent surgery; meanwhile, patients with unresectable advanced, recurrent, or metastatic GC were excluded, in order to obtain accurate results; the patients with a transregional tumor were also excluded. Because R2 resection indicates gross (macroscopic) residual disease, it should be classified as a palliative resection. Therefore, patients who underwent R2 resection were also excluded. Moreover, the effect of molecular subtypes and *H pylori* infection on the clinicopathology of GC was not investigated. Owing to these limitations, our results are not applicable to all patients with GC. Hence, further study is necessary.

In conclusion, our results suggest a clinicopathological difference between GC in the lesser curvature and GC in the greater curvature. These findings support the distributing characteristics of GC and contribute to the development of appropriate treatment for GC.

## Acknowledgments

All authors would like to thank Dr Man Guo and Dr Xiao Lian for their help in the design, data collection, and analysis.

## Author contributions

Li GC conceived and designed the study, and wrote the manuscript; Zhang HW contributed to the critical revision of this manuscript; Tian HG, Zhao Y, and Huang QX analyzed and interpreted the data; Xu ZY, Xi XH, and Zhang K contributed to the collection of related literatures. All authors approved the final version of the manuscript.

## References

[1] Fitzmaurice C Allen C Barber RM; Global Burden of Disease Cancer Collaboration. Global, regional, and national cancer incidence, mortality, years of life lost, years lived with disability, and disability-adjusted life-years for 32 cancer groups, 1990 to 2015: a systematic analysis for the global burden of disease study. JAMA Oncol. 2017;3:524–48. 27918777 10.1001/jamaoncol.2016.5688 PMC6103527

[2] Allemani C Weir HK Carreira H. Global surveillance of cancer survival 1995-2009: analysis of individual data for 25,676,887 patients from 279 population-based registries in 67 countries (CONCORD-2). Lancet. 2015;385:977–1010. 25467588 10.1016/S0140-6736(14)62038-9 PMC4588097

[3] Morgagni P Solaini L Framarini M. Conversion surgery for gastric cancer: a cohort study from a western center. Int J Surg. 2018;53:360–5. 29654967 10.1016/j.ijsu.2018.04.016

[4] Cancer Genome Atlas Research N. Comprehensive molecular characterization of gastric adenocarcinoma. Nature. 2014;513:202–9. 25079317 10.1038/nature13480 PMC4170219

[5] Birkman EM Mansuri N Kurki S. Gastric cancer: immunohistochemical classification of molecular subtypes and their association with clinicopathological characteristics. Virchows Arch. 2018;472:369–82. 29046940 10.1007/s00428-017-2240-x PMC5886993

[6] Liu X Meltzer SJ. Gastric cancer in the era of precision medicine. Cell Mol Gastroenterol Hepatol. 2017;3:348–58. 28462377 10.1016/j.jcmgh.2017.02.003 PMC5404028

[7] Kim HJ Kang SK Kwon WS. Forty-nine gastric cancer cell lines with integrative genomic profiling for development of c-MET inhibitor. Int J Cancer. 2018;143:151–9. 29435981 10.1002/ijc.31304

[8] Hansford S Kaurah P Li-Chang H. Hereditary diffuse gastric cancer syndrome: cdh1 mutations and beyond. JAMA Oncol. 2015;1:23–32. 26182300 10.1001/jamaoncol.2014.168

[9] Eom BW Jung KW Won YJ. Trends in gastric cancer incidence according to the clinicopathological characteristics in Korea, 1999-2014. Cancer Res Treat. 2018;50:1343–50. 29361823 10.4143/crt.2017.464 PMC6192902

[10] Cristescu R Lee J Nebozhyn M. Molecular analysis of gastric cancer identifies subtypes associated with distinct clinical outcomes. Nat Med. 2015;21:449–56. 25894828 10.1038/nm.3850

[11] Sheikh IA Mirza Z Ali A. A proteomics based approach for the identification of gastric cancer related markers. Curr Pharm Des. 2016;22:804–11. 26648471 10.2174/1381612822666151209151848

[12] Zhou H Dong A Xia H. Associations between CA19-9 and CA125 levels and human epidermal growth factor receptor 2 overexpression in patients with gastric cancer. Oncol Lett. 2018;16:1079–86. 29963185 10.3892/ol.2018.8731 PMC6019918

[13] Laboissiere RS Buzelin MA Balabram D. Association between HER2 status in gastric cancer and clinicopathological features: a retrospective study using whole-tissue sections. BMC Gastroenterol. 2015;15:157. 26530403 10.1186/s12876-015-0384-1 PMC4632681

[14] He C Bian XY Ni XZ. Correlation of human epidermal growth factor receptor 2 expression with clinicopathological characteristics and prognosis in gastric cancer. World J Gastroenterol. 2013;19:2171–8. 23599643 10.3748/wjg.v19.i14.2171 PMC3627881

[15] Clavien PA Barkun J de Oliveira ML. The Clavien-Dindo classification of surgical complications: five-year experience. Ann Surg. 2009;250:187–96. 19638912 10.1097/SLA.0b013e3181b13ca2

[16] You WC Zhang L Gail MH. Gastric dysplasia and gastric cancer: Helicobacter pylori, serum vitamin C, and other risk factors. J Natl Cancer Inst. 2000;92:1607–12. 11018097 10.1093/jnci/92.19.1607

[17] Cho SJ Choi IJ Kook MC. Randomised clinical trial: the effects of Helicobacter pylori eradication on glandular atrophy and intestinal metaplasia after subtotal gastrectomy for gastric cancer. Aliment Pharmacol Ther. 2013;38:477–89. 23822578 10.1111/apt.12402

[18] Hayashi S Imamura J Kimura K. Endoscopic features of lymphoid follicles in Helicobacter pylori-associated chronic gastritis. Dig Endosc. 2015;27:53–60. 25092073 10.1111/den.12335

[19] Okamoto N Kawachi H Yoshida T. “Crawling-type” adenocarcinoma of the stomach: a distinct entity preceding poorly differentiated adenocarcinoma. Gastric Cancer. 2013;16:220–32. 22865191 10.1007/s10120-012-0173-2

[20] Galizia G Lieto E Auricchio A. Comparison of the current AJCC-TNM numeric-based with a new anatomical location-based lymph node staging system for gastric cancer: a western experience. PLoS One. 2017;12:e0173619. 28380037 10.1371/journal.pone.0173619 PMC5381862

[21] Asakawa Y Ohtaka M Maekawa S. Stratifying the risk of lymph node metastasis in undifferentiated-type early gastric cancer. World J Gastroenterol. 2015;21:2683–92. 25759537 10.3748/wjg.v21.i9.2683 PMC4351219

[22] Griniatsos J Moris D Spartalis E. Towards a tailored lymphadenectomy for gastric cancer based on the correlation between the primary tumor location and the first lymphatic drain basin: preliminary data. J BUON. 2017;22:1137–43. 29135094

[23] Aoyama J Kawakubo H Goto O. Potential for local resection with sentinel node basin dissection for early gastric cancer based on the distribution of primary sites. Gastric Cancer. 2019;22:386–91. 30099636 10.1007/s10120-018-0865-3

[24] Lee JH Lee HJ Kong. SH. Analysis of the lymphatic stream to predict sentinel nodes in gastric cancer patients. Ann Surg Oncol. 2014;21:1090–8. 24276637 10.1245/s10434-013-3392-9

[25] Hirota M Nakajima K Miyazaki Y. Clinical outcomes of laparoscopic partial gastrectomy for gastric submucosal tumors. Asian J Endosc Surg. 2015;8:24–8. 25470074 10.1111/ases.12145

[26] Kim M Jeon SW Cho KB. Predictive risk factors of perforation in gastric endoscopic submucosal dissection for early gastric cancer: a large, multicenter study. Surg Endosc. 2013;27:1372–8. 23239296 10.1007/s00464-012-2618-4

[27] Zhu YL Yang L Sui ZQ. Clinicopathological features and prognosis of Borrmann type IV gastric cancer. J BUON. 2016;21:1471–5. 28039710

[28] Kang WM Meng QB Yu JC. Factors associated with early recurrence after curative surgery for gastric cancer. World J Gastroenterol. 2015;21:5934–40. 26019458 10.3748/wjg.v21.i19.5934 PMC4438028

[29] Ajani JA Mayer RJ Ota DM. Preoperative and postoperative combination chemotherapy for potentially resectable gastric carcinoma. J Natl Cancer Inst. 1993;85:1839–44. 8230264 10.1093/jnci/85.22.1839

[30] Kang SH Kim YH Roh YH. Gallstone, cholecystectomy and risk of gastric cancer. Ann Hepatobiliary Pancreat Surg. 2017;21:131–7. 28989999 10.14701/ahbps.2017.21.3.131 PMC5620473

[31] Aratani K Komatsu S Ichikawa D. Overexpression of EGFR as an independent prognostic factor in adenocarcinoma of the esophagogastric junction. Anticancer Res. 2017;37:3129–35. 28551654 10.21873/anticanres.11670

